# Comparative results of non-operative multi-modal therapy for filarial lymphoedema

**DOI:** 10.4103/0970-0358.53008

**Published:** 2009

**Authors:** S. B. Gogia, N. C. Appavoo, A. Mohan, M. Burney Kumar

**Affiliations:** Sanwari Bai Surgical Centre, 28/31 Old Rajinder Nagar, New Delhi - 110 060, India; 1 Director of Public Health, Directorate of Health Services, Tamil Nadu, India; 2 Filaria Therapy Control Unit, Chengalpattu Dist, Tamil Nadu, India

**Keywords:** Filariasis, Heat therapy, Interferential therapy, Lymphoedema, Pneumatic compression

## Abstract

A comparative analysis of different conservative modes of therapy for lymphoedema, largely of Filarial origin, was conducted in a trial therapy unit in Chengalpattu, a Filarial endemic district in Tamil Nadu. Results were compared using a single chambered intermittent pneumatic compression pump, heat therapy, and interferential therapy machines. The results showed improvement of limb size between 20% and 60% of possible reduction (where 100% would mean return of limb circumference to the same as that of the normal side). Pneumatic compression therapy, when used alone, showed the best results, which were significantly better than all others whether alone or in combination.

## INTRODUCTION

Lymphoedema is a common clinical problem in our country. It is more prevalent in our coastal areas. Its treatment strategies have not yet been well defined. Very little work has been done in our country especially towards mitigating the resulting disfigurement.

Recently, some claims propagating heat therapy, compression therapy, and interferential therapy have been made and the requisite equipment had been presented in the first annual meeting of the Indian Society of Lymphology at Thanjavur in 1996. Since the state government was planning mass therapy trials of annual mass single-dose administration of DEC salts, a lymphoedema therapy trial unit was set up in Chengalpattu in 1996 to try the various modalities available and to formulate a strategy for mass treatment for the victims in the state of Tamil Nadu. Based on a review of literature and discussion with experts at this meeting, a few protocols for treatment of patients with filariasis were prepared to provide a morbidity control adjunct. The initial results of this study are being presented.

## MATERIAL AND METHODS

The lymphoedema therapy trial unit in Chengalpattu is located in the premises of an old disused hospital. The center is equipped with a) a Heat Therapy machine, b) a Pneumatic Compression therapy unit, and c) an Interferential Therapy (IFT) machine. The staff is well trained in application of all three modalities. A well appointed airy ward was set aside for the treatment center. A doctor initially saw the patient and allocated the cases to various protocols as per the current ongoing trial. Case selection was by randomization but was not double-blind.

All the modalities were available in the same premises and run by a set of paramedical workers who had been provided training in administering all of them. They also took measurements, reported the progress as well as gave initial advice for safety measures and possible complications to the patients. Treatment was continued until the weekly measurements showed no further improvement in the results or if the patient chose to terminate the treatment.

### Ancillary treatment (common to all the groups)

All patients were given training in self massage. Foot care and thorough cleaning was emphasized. Lympedim^®^ (Coumarin 200 mg - M/s Pharm Products, Thanjavur, Tamil Nadu, India) tablets were provided free of cost to all. Compressive bandaging using a crepe bandage was done in between therapy periods for patients who could afford to purchase the same.

Circumference measurement at various prefixed points were taken on both sides initially and then onwards on the involved side weekly until the end of therapy. The measurements were taken by a trained para medical worker using a specially provided tape and entered into the patients chart [Table 1].

**Table 1:** Sample measurement chart

*Pos Date*		*Left 28/4/00*		*Right 28/4/00*	
D				
C				
B				
A				
0				
1				
2				
3				
4				
5				
6				
7				
8				
9				
10				
11				
12				
13				
14				
15				

The following treatment modalities were tested.

1. **Heat therapy** – The affected limb of the patient is inserted for 30–45 minutes in a special chamber that provides radiant heat through 16 100-watt electric bulbs (“M/s ELECTROCARE”). The temperature can be thermostatically controlled to between 60° and 70° according to patient tolerance.
2. **Pneumatic compression therapy** – A VIPEL^®^ (*M/s AMLA MEDIQUIP*) apparatus was used. The involved limb is inserted in a single-chambered encasing that is inflated to between 80 and 160 mm Hg pressure. The pressure is made to rise slowly to peak in about 120-180 seconds and then a rest period (0 mm Hg pressure) of between 30-45 seconds is provided by an electronic circuit. Therapy was continued for 2-3 hours per day and then a crepe bandage was applied.
3. **IFT therapy + heat** – In IFT, a faradic current of 15–30 millivolts is passed for 30 minutes in 5 second pulses in selected points of the involved limb. Heat therapy was also given as well as compressive bandaging.
4. **Combined therapy** – All patients are administered heat therapy (2 weeks), pneumatic compression (2 weeks), and IFT (2 weeks) in a sequential manner.

In the initial phase of our study, all patients were being allocated to combined therapy (number 4 above). Later, once experience of the various modalities was available with the staff, a decision was made to make a proper trial and patients thereafter were allocated to individual groups i.e., numbers 1, 2, and 3 above (Table 2 for the group-wise allocation of the patients).

**Table 2:** Overall results (opposite side measurement used for patients with B/L edema)

	*Treatment time (months)*			*Total reduction (cm)*			*Average redn (cm)*		
			
	*Total (of all)*	*Max*	*Avg*	*Inv. side*	*Opp side*	*Avg (%)*	*Inv. side*	*Opp side*
Heat + IFT (18 limbs)	131	18	8.39	15	0.5	23.8	0.83	0.03
Combined (43 limbs)	331	18	8.71	38	7	18.9	1.01	0.18
Compression (31 limbs)	98	18	3.94	72	4.5	44.3	2	1.13
Heat alone (58 limbs)	377	14	6.61	49.5	17.5	18.3	1.35	0.87

Treatment was continued for others as long as the patient returned or it was felt that no further treatment was required i.e., the weekly measurements had reached a plateau.

**Figure 1:**
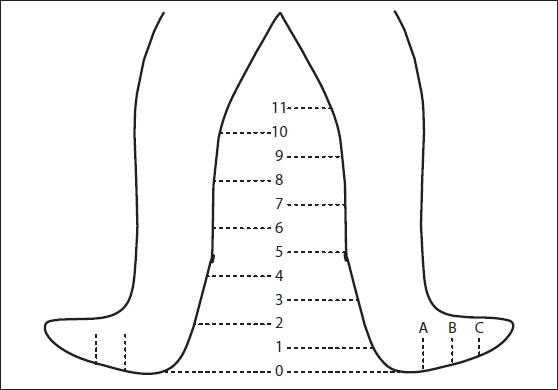
Measurement points in the legs

Results were tabulated as follows: The limb was initially marked heel upwards to create points 1, 2, 3, etc. at 6 cm apart from the previous mark [Figure 1] e.g., the heel point is point 0 and point 1 is 6 cm superior. Similarly points A, B, C, and occasionally D were created at distances 4.5 cm distally from point 0. The knee was marked with a K for later confirmation that the repeat measurements were accurate i.e., if the current K is not where previously shown, it meant that the previous measurements do not match the current measurements.

An individual chart was prepared for each patient [Table 1] where circumferences at these points were noted for both sides initially and then weekly thereafter until the end of therapy for the involved side. The circumference that showed the maximum difference between the two limbs was selected for the calculation of results. The initial difference was compared with the final difference and the improved circumference results calibrated as a percentage of the possible (i.e., 100% if the improvement was equal to the initial difference). The same method was followed in all the different treatment modalities. Patients with disease on both sides were excluded from the final calculations of the study. The results were evaluated using Student's *t* test through a computer.

## RESULTS

The results are shown in the accompanying Tables Tables 2–4. Table 2 shows the total number of evaluable limbs of patients who completed therapy for each group. A comparison of normal with abnormal limbs precluded presentation of a comparative analysis in all patients as some had bilateral disease. Thus, results are available for fewer patients than the total. Most of the patients with bilateral disease were from Group 3 (Heat + IFT) so that group has a fewer number of patients in the final analysis.

**Table 4:** Statistical analysis

*Area earmarked with shaded background shows significance*	*Standard deviation*	*Probability using t-test*			
	
		*With heat*	*Compression*	*Combine*	*IFT + H*
Heat	0.4061	----	0.0493	0.6843	0.8665
Compression	0.3933	0.0493	----	0.0496	0.1971
Combined therapy	0.5043	0.6843	0.0496	----	0.9225
Heat + IFT	0.8449	0.8665	0.1971	0.9225	----

All patients tolerated the treatment sessions well and completed their regimes without break. Treatment sessions averaged between 3–6 months with a maximum of 18 months. No major complications were reported. Table 3 shows the results obtained.

**Table 3:** Improvements

*Therapy type*	*Number of patients with improvement (% of total)*				*Total evaluable limbs*	*Average redn (comparative)*	
		
	*>75%*	*Between 50% and 75%*	*Between 25% and 50%*	*<25%*		*(Cm at max point)*	*% of possible*
Heat + IFT	2 (28.6)	0 (0)	2 (28.6)	3 (42.9)	7	0.7	38.92
Combined	4 (14.4)	7 (26.9)	1 (3.9)	14 (53.9)	28	0.76	21.74
Compression	8 (34.8)	6 (26.1)	3 (13.0)	6 (26.1)	25	1.91	58.13
Heat	7 (21.1)	6 (18.2)	4 (12.1)	16 (48.5)	43	0.99	31.41

The results showed a marginally significant difference between heat therapy alone and compression therapy. Compression therapy is better (*P* < 0.05). There is also a significant (*P* < 0.05) difference between combined therapy and compression alone favouring the latter. The number of patients in the Heat + IFT group were too few for a significant result to be obtained in comparison with the other modes.

## DISCUSSION

Lymphoedema is a common clinical problem in India, as lymphoedema is the most common clinical manifestation of filariasis.[1] There exists a vast number of patients afflicted by this crippling disease. The magnitude of this problem can be seen from the fact that nearly 200 million people (20% of the population) live in the endemic zone and more than 19 million suffer from the disease.[2]

### Aetiopathogenesis

Lymphoedema is caused by a relative mismatch between lymphatic load (the amount of lymph formed) and lymphatic transport capacity (i.e., a cross-section of lymph vessels multiplied by lymph flow.)[3] This may be due to overproduction of lymph (e.g., congestive cardiac failure, hypoproteinemia, or venous block) or due to decreased lymphatic flow (lymphatic obstruction, filariasis).[4]

This causation is of relevance to therapy as most forms of conservative therapy depend on the availability of some lymphatics. In filariasis, the initial problem caused by the worm is early lymphatic dysfunction and dilatation of the lymph vessels.[5] At an early stage, the edema may be reversible.[5] Occurrence of lymphoedema has been correlated with 1) the incidence of microfilaraemia, 2) immunity especially cellular toxicity to the microfilaria, and 3) age, which may be dependent on development of immunity.[6,7]

Lymphatics are capable of regeneration so a simple obstruction is incapable of producing lymphoedema. Recovery is due to regeneration of the lymphatics, but may not occur if development of collaterals is inhibited.[8] The culprit here is mostly secondary infection generally of streptococcal origin. Lymphoedema has also been described as continuing inflammation.[9] When lymph flow is blocked, as also in inflammation, the leaked tissue protein is immediately precipitated into a fibrinous network.[10] Fibroblasts wander into the area leading to a thickening of subcutaneous tissue and multiplication of collagenous elastic fibers, thus resulting in non pitting edema in late cases.[11,12] In early stages, the edema may be reversible dependent on grading.[11]

Attacks of streptococcal lymphangitis have a major role in the precipitation of clinical lymphoedema as the resulting inflammation causes further obstruction of the lymphatic system.[13] Lymphangitis attacks are described to occur more frequently in secondary forms of lymphoedema, notably of filarial origin.

Any swollen limb, the initial cause of swelling being cardiac, hepatic, filariasis, paralysis, etc., is more prone to secondary streptococcal infection. We have seen secondary infection in paralysis cases too. Once the infection sets in, it becomes self-perpetuating. Severe cellulites occurring de novo may also be the primary offending episode. That is why antibiotics (especially penicillin) have such an important role in management.

China, where the incidence of filariasis has fallen due to its salt fortification with Diethyl Carbamezine (DEC), has seen a decrease in chronic manifestations.[14] Between 1995 and 1997 and 2003 and 2005, many districts in Tamil Nadu have seen a fall in microfilaremia (mf) incidence. This has been most notable in the district of Kanyakumari where the DEC fortification in salt program took place. We expect a similar fall in incidence of chronic manifestations in such other places in India over time.

In late cases, following recurrent inflammatory attacks, the skin also becomes thickened and may show papillomatosis, excrescenses, verrucal changes, recurrent ulceration, and hyperpigmentation finally ending in a non pitting fibrotic solid edema.[5] Most thickening and tissue distension is found in the dermis and subcutaneous tissue. However, some increase in the muscle bulk has also been described by ultrasound studies. An increase of up to 75% in cases of primary lymphoedema and up to 50% in secondary lymphoedema has been seen.[15] In the same study, the average thickness of the dermis was 3–4 mm (normal 1–2 mm). The bulk of the increase was, however, in the subcutaneous tissue varying between 21–79% of normal.

The basis of our conservative therapy is the belief that** lymphoedema is continuing inflammation. All modes used in this study were actually anti-inflammatory agents as our further discussions show.

### Intermittent pneumatic compression

In 1948, Joseph Conrad E., an engineer, noted that the conventional elastic stocking used in treatment of venous edema resulted in more folds of the skin rather than convenience to the patient. Later, he found that standing in a swimming pool decreased edema and made the patient more comfortable. He attributed this to the uniform pressure exerted by water. This lead him to manufacture the Jobst elastic stocking, which took into account all the natural folds and contours of the limb. Later, in 1951, he invented an extremity pump that could drive out fluid from the extremity.[16] Since then, intermittent pneumatic compression (IPEC) using a mechanical pump [Table 5], has been found useful in cases of mild to moderate lymphoedema.[15,17]

**Table 5:** Comparing sequential with simple pumps

	*Sequential*	*Simple*
Time per cycle	30 seconds	3-4 Minutes
Average inflation time per chamber	<3 seconds	60-90 seconds
Peak pressure obtained	100 mm Hg	180 mm Hg
Number of chambers	12	1
Average pressure increment	35 mm Hg/sec	3 mm Hg/sec
Average time of peak pressure	10 seconds	60 seconds

The principle of this pump is as follows: The limb is covered by a plastic inflatable encasing and connected to an electrically operated air pump. A series of valves in the circuit are pre-timed to inflate the encasing with air for a period of 90–180 seconds, causing increased pressure circumferentially around the limb. This compression pressure reaches, and sometimes crosses the systolic artery pressure. Pressure is then turned off for a period of 20–40 seconds to restore limb circulation.

A ‘massaging’ pump in which pressure is applied in a wave form to the limb has been described. The pressure waves travel centripetally.[18] An improvement of 37%, 41%, and 31% in the dorsum, leg, and upper portions, respectively with Lymphapress® has been reported.[19] These are slightly lower figures than our results, which are 45% overall reduction and 58% at the point of maximum circumference.

The suggested mode of action of IPEC[20] is that it increases interstitial fluid pressure so that water is forced hydrostatically across the capillary bed into the venous system. The resulting increase in venous outflow increases further clearance of water and colloid. Existing lymphatic and collateral flow is also augmented in a similar fashion. Tissue compressibility or compliance affect the pressure head that can develop in the subcutaneous tissue. Fibrotic or ligneous tissue is less compressible, so in patients with advanced fibrosis, the massaging effect of the pump is not so effective. This results in decreased fluid clearance via the lymphatic collaterals or across the tissue capillaries. Severe venous obstruction and severe subcutaneous fibrosis (Grade III) have been suggested as contraindications to IPEC.

However, we still do not know why mechanical massage works; though few do recommend it[21] while some are vehemently against it.[22] Our perceived mode of action is different and is explained below as well as in the accompanying box No. 1.

**Figure 2:**
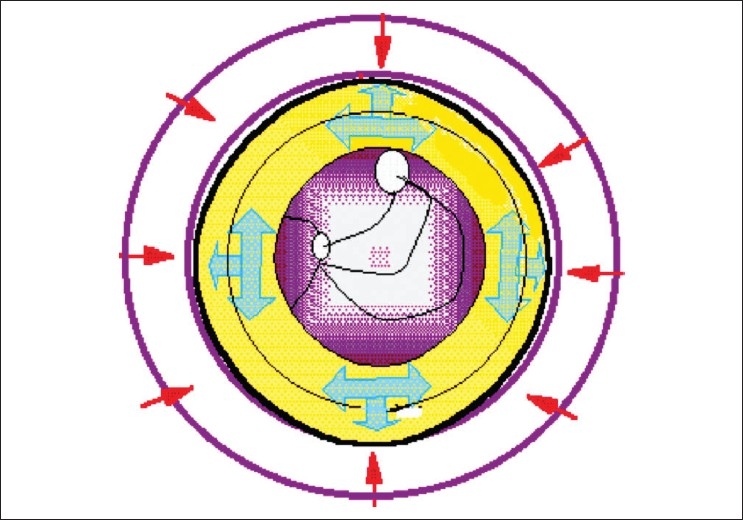
Arch Effect. Circumferential pressure (red arrows) is absorbed and spread circumferentially (thin black circle) along the line of maximum resistance (blue arrows)

**Box 1:** Equations regarding the relationship of pressure and heat. Maximum pressure is absorbed by the Area of Fibrosis in Lymphoedema [Arch Effect - Figure 2]. Heat and pressure likely have a common pathway of effectiveness. However, pressure generates better heat in the required area (area of fibrosis) and hence has a better effect. Radiant heat (given in our cases) acts on the outer surface. A sudden rise in pressure, which occurs in sequential pumps (as apposed to a graduated rise), may be harmful especially if a wave is generated to push the fluid manually

“External compression causes pressure on the tissues. This pressure rise causes an increase in available energy in the tissues due to a decrease in volume. Thus, pressure results in a rise of localized temperature.
[t1 - t2 : t2 - t1 = + ˆt].
Rise in temperature causes an increase of biological entropy i.e., the relation between various molecules like proteins, Na^+^, Mg^+^, fats etc.”
“Entropy change may be defined as the amount of heat absorbed isothermally and reversibly divided by the absolute temperature.
ˆS = q/T
Where ˆS = change in entropy
T = Absolute temperature
&q = amount of extra heat available due to pressure.
Every system tends to minimize free energy via entropy.
Gibbs Free Energy
G = H - TS
where H = Heat Content
T = Absolute temperature
&S = Entropy
∴ˆG = ˆH - TˆS
Thus, a decrease in free energy of a particular system is a measure of the useful or net work during the change.
*Increase of tissue temperature and available tissue heat releases pyrogens from the WBCs. This activates the macrophages to eat up extracellular fluid protein and restore the negative tissue pressure status.*
(Another simpler equation would be based on the Universal Gas Equation or Avagadro's Law i.e.,
$\frac{\mathrm{PV}}{T}$
where **P** = Pressure
**V** Volume and
**T** Temperature
Hence Pressure at constant Volume raises the Temperature)
For more components to the universal gas equation, please see http://www.mikeblaber.org/oldwine/chm1045/notes/Gases/IdealGas/Gases04.htm)

Unilocular compression compresses the limb circum-ferentially. Most of the pressure head is absorbed by the ring of circumferential fibrosis occurring in lymphoedema due to an arch effect [Figure 2]. There is slowly rising gradual compression of the limb that finally maintains the peak pressure for about 30 to 60 seconds (the rest of the time is spent in increasing the pressure from 0). During “Off” time the pressure decreases to 0. Compressed tissue generates - heat (see the explanation in box No. 1) and probably causes a similar action as a heat therapy machine. Tissue heating (in theory, based on this hypothesis) by compression would be maximum at the place of maximum fibrosis. While the heat therapy machine used in this study creates maximum heat at the epidermis, a microwave oven would generate uniform heating of all the tissues - even of the muscles. The significantly better results in our study favor this theory, but a proper detailed study is awaited.

Most available studies on pneumatic compression have been on primary or post-mastectomy edema. In the latter, up to a 50% reduction has been described in 18 out of 25 cases by Bunce,[23] while Wozniewski[24] described a 50%, 40%, and 25% reduction in mild, moderate, and severe cases, respectively. Our cases had a higher inflammatory component than the above studies, one of the reasons behind our better results.

A question mark has arisen regarding the use of high pressure and also the use of unilocular (as apposed to sequential) compression. The main objection provided is that it is compression of the lower end and could not physiologically push fluid out of the swelling.[25] Miller and Seale[26] used weights to load the limb. They found that lymph clearance increased until 60 mm Hg and then declined sharply so that after 75 mm Hg there was no flow. In filarial patients, better results than with the sequential pump have been obtained using higher pressures with no problems.[27] We believe that higher pressures were the reason for their results of a 65% average decrease in the limb size.

**Figure 3:**
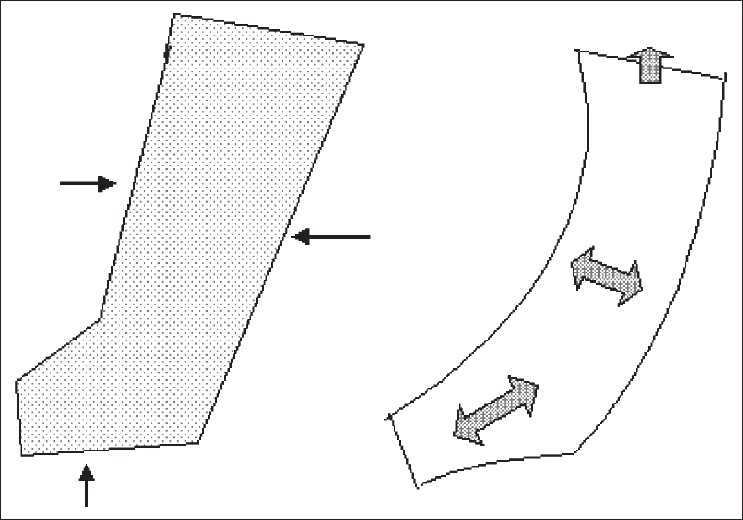
The single chambered pump can only move fluid up near its upper margin. Pressure is applied gradually

**Figure 4:**
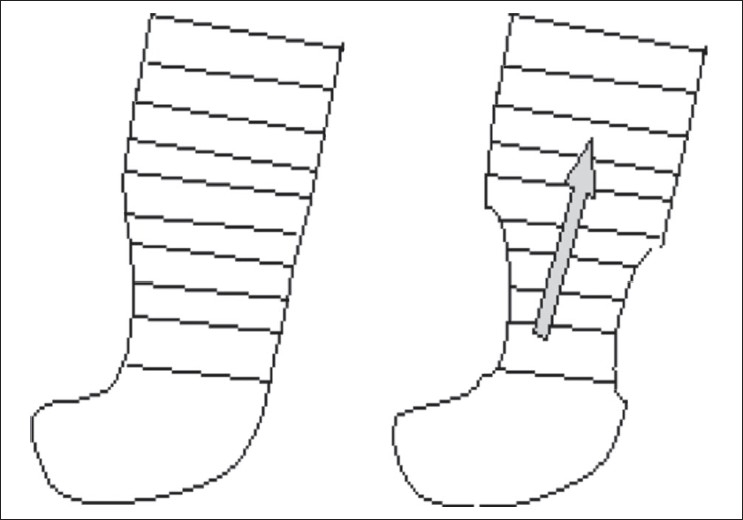
The sequential pump moves fluid directly in an upward direction due to fast

We also do not believe that simple pressure just pushes fluid up - unlike sequential pressure [Figures 3 and 4]. Use of the sequential pump has also resulted in a high incidence of problems such as thigh swelling, scrotal swelling, and also swelling going across to the uninvolved side. Increased overload of the cardiac and renal systems especially in compromised patients has also been reported.[21,23] We believe that, at least for the management of filarial lymphoedema, unilocular compression gives good results without problems.

Manual compression by a trained physiotherapist[28,29] is agreeably the preferred mode but may be unsuitable in our country due to the large number of cases and relative lack of trained staff to administer the therapy.

Our results actually seem better but that may not be so, as we have used a different measurement for improvement. We have targeted improvement in the maximum site while others have used average improvement. We believe that a uniformly contoured leg is more desirable and is also important for proper fitting of a garment. Average improvement is more difficult to measure especially when the thigh or dorsum is the involved segment for the following reasons:

- The change in shape may not correlate with improve-ment as the circumference is what is being measured (we had not used volume measurements)
- The entire limb is not always involved, sometimes even a partial segment of a portion (especially the thigh) has swelling. The average generally includes normal areas, which if included in the final measurements will show less improvement than there would be.

### Heat treatment

The Chinese have evolved their own method of treatment of lymphoedema. This traditional therapy was associated with patients putting their affected limbs in household ovens (woks) once they were back from work at the end of the day.[30] Therapy consisted of an intense delivery of heat to the edematous tissue. Previous use of electric heating or warm bandaging has been replaced with microwave therapy in the country of its origin.[31] This has yielded 75% good or excellent results with up to 3 courses of 15 days each. Each therapy session lasts for around 30 minutes in the specially designed oven. However, long-term results were not described. Temperatures of approximately 40° are generated in the muscles and subcutaneous tissues. Elastic dressing is helpful as a adjunct.

**Figure 5:**
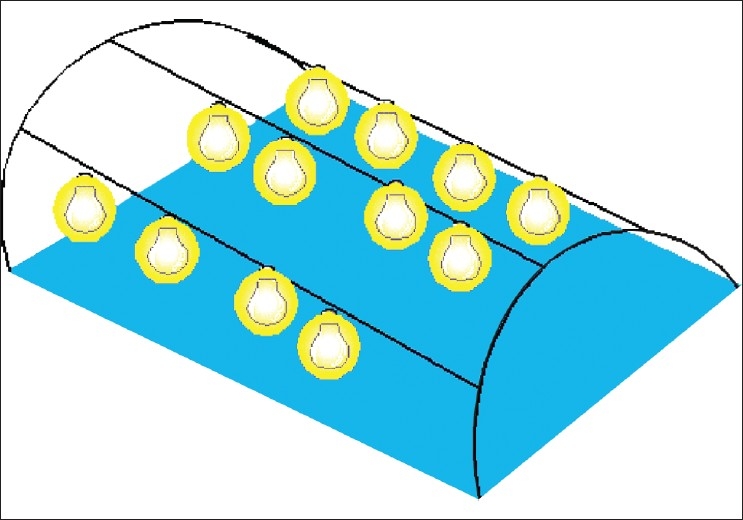
Heat therapy machine - patients legs are enclosed in a dome heated by sixteen 100 Watt bulbs (outer cover not shown for clarity). Since Indian skins are darker, the heat may be absorbed at the epidermal level. Hence patient tolerance was lower and beneficial effect may be less

We had used a simple indigenous apparatus [Figure 5] developed by Dr. Tambwekar,[29] a senior Plastic Surgeon in Mumbai. He has been using this apparatus for many years at Mumbai. It gives radiant heat to limb; arguably less effective than microwave, as heat does not reach the required sub-dermal plane without burning the epidermis. An average reduction of 31% was achieved with associated softening of skin texture in our patients. Dr. Tambwekar has also designed a simple and economical elastic bandage that has velcro all along to disallow slippage. That was used in those of our patients who were willing to purchase it.

Olszewski[19] has described this therapy as useful in some cases of post surgical and post inflammatory lymphoedema.

### Interferential therapy

This is a physiotherapy machine where a faradic current of 15–30 millivolts is passed for 15 minutes in 5-second pulses to selected points of the involved limb. The mode of action is yet unknown, but may be related to heat generation. Good results have been reported after this study in mild lymphoedema but a lack of definite work on this modality of treatment prevented us from using it as the sole therapy in the current study.

In conclusion, the results show a marginally significant difference between heat therapy alone and compression therapy. Compression therapy is better (*P* < 0.05%). There also exists (*P* < 0.05%) difference between combined therapy and compression alone. Since fewer patients came under the IFT + heat group, these results do not appear significant.

We are sanguine that the availability of conservative treatment modalities should be widely recommended for use in all the filariasis affected districts in our country. With an overwhelmingly large population of patients with lymphoedema in our country, it makes eminent sense to have therapy freely available in endemic areas.

Besides, we have been treating lymphoedema at our own clinic. We have seen patients come back with recurrence as well as worsening of symptoms; but find most incidences related to adenolymphangitis (ADL) attacks. ADL attacks have been shown to decrease following pneumatic compression[27] as well as through ancillary measures alone.[32] More important than the compression or any other edema therapy is proper limb cleaning, regular antibiotics (especially penicillin) and other measures to prevent ADL attacks.

We do not have adequate numbers of trained staff for providing manual drainage[33] to the vast number of afflicted patients in our country. We have come to believe that the above machines offer a possible alternative as they are cheap, can be made available, and do not require very highly trained workers.

The results of this study have already been applied on a wider scale to create filaria treatment centers in over 70 centers in the district of Tamil Nadu where the above cocktail of therapies is now available.
